# *Rickettsia sibirica mongolitimonae* Infection, France, 2010–2014

**DOI:** 10.3201/eid2205.141989

**Published:** 2016-05

**Authors:** Emmanouil Angelakis, Herve Richet, Didier Raoult

**Affiliations:** Aix Marseille Université, Marseille, France

**Keywords:** Rickettsia sibirica mongolitimonae, skin biopsy, cutaneous swab, rope-like lymphangitis–associated rickettsiosis, rickettsia, bacteria, France, vector-borne infections

## Abstract

To further characterize human infections caused by *Rickettsia sibirica mongolitimonae*, we tested skin biopsy and swab samples and analyzed clinical, epidemiologic, and diagnostic characteristics of patients with a rickettsiosis. The most common (38%) indigenous species was *R. sibirica mongolitimonae*. Significantly more cases of *R. sibirica mongolitimonae* infection occurred during spring and summer.

Tickborne rickettsioses are zoonoses caused by spotted fever group (SFG) *Rickettsia* spp. (*1*). The first human infection with *R. sibirica mongolitimonae* was reported in France in 1996 (*2*). This patient had rope-like lymphangitis from the eschar to the draining lymph node, and *R. sibirica mongolitimonae* infection was thus named lymphangitis-associated rickettsiosis (*3*,*4*). Since then, other cases with or without rope-like lymphangitis have been described (*5*). Several SFG rickettsioses that have been considered nonpathogenic for decades are now associated with human infections, making these diseases useful as a paradigm for understanding emerging and reemerging infections (*6*). To further characterize human infections caused by *R. sibirica mongolitimonae*, we tested skin biopsy and swab samples and analyzed the clinical, epidemiologic, and diagnostic characteristics of patients with a rickettsiosis.

## The Study

During 2010–2014, we tested skin biopsy (*7*) and cutaneous swab samples from rickettsiosis inpatients and outpatients throughout France. These samples were received frozen or in transport media; when possible, serum samples were also collected and sent at room temperature. For patients with positive *Rickettsia* results, epidemiologic and clinical data were collected.

We extracted total genomic DNA from samples by using a QIAamp tissue kit (QIAGEN, Hilden, Germany). We screened samples for *Rickettsia* spp. by using a quantitative PCR assay selective for a 109-bp fragment of a hypothetical protein (*8*). For positive samples, PCR amplification and sequencing selective for the *glt*A and *omp*A genes were performed (*8*). Samples were cultured in human embryonic lung fibroblasts (*9*). All serum samples were tested by immunofluorescence assay for SFG rickettsial antigens and typhus group rickettsiae (*10*). Student *t* or χ^2^ tests were performed by using Epi Info version 6.0 software (Centers for Disease Control and Prevention, Atlanta, GA, USA). Means were compared by using analysis of variance or the Kruskal-Wallis test, on the basis of results of the Bartlett test for inequality of population variances. Proportions were compared by using the Mantel-Haenszel χ^2^ or Fisher exact tests when the expected value of a cell was <0.05. *R. sibirica mongolitimonae* seasonality was assessed by using the autocorrelation module of PASW software version 17.02 (http://www.spss.com.hk/statistics/). p <0.05 was considered significant.

We classified patients as definitively having a rickettsiosis if direct evidence of rickettsial infection was found on culture or molecular assays. Of 465 patients examined, 91 (20%) were infected with *Rickettsia* spp., most commonly *R. africae* (n = 36, 40%), followed by *R. conorii* (n = 21, 23%), *R. sibirica mongolitimonae* (n = 20, 22%), and *R. slovaca* (n = 14, 15%). Two cases of *R. sibirica mongolitimonae* infection in France have been reported (*11*,*12*).

For patients infected with *R. sibirica mongolitimonae*, median age ± SD (interquartile range) was 43 ± 21 (2–70) years, and most (12, 60%) were male (Technical Appendix Table 1). The most common *Rickettsia* species in France was *R. sibirica mongolitimonae.* Only 1 patient mentioned recent travel to Spain; all others denied recent travel. Five patients mentioned recent outdoor activities, 8 mentioned frequent contact with dogs, and 1 mentioned contact with horses. A tick bite or tick handling was reported by 6 patients. An autocorrelation analysis revealed significant seasonality for *R. sibirica mongolitimonae* cases (p<0.001). Significantly more cases occurred during spring (April–June) (11 cases, 55%; p = 0.006), followed by summer (July–September) (8 cases, 40%, p = 0.01). One case occurred in October and none in winter.

The symptoms at disease onset included fever for all patients (duration 4–14 days), myalgia (n = 11, 55%), and headache (n = 3, 15%). Generalized maculopapular rash and an inoculation eschar developed in all patients. One patient had 3 eschars (buttocks, right hand, breast). A rope-like lymphangitis from the eschar to the draining lymph node was detected in 7 (35%) patients. One patient was admitted to an intensive care unit. For all 5 patients for whom an initial laboratory examination was available, increased liver enzymes (alanine aminotransferase, aspartate aminotransferase) and thrombocytopenia were found; 2 patients had hypoproteinemia. Oral doxycycline (7–14 days) was given to 19 patients; pristinamycin (7 days) was given to 1 patient. All outcomes were successful.

An eschar swab sample was available for 13 patients (*13*), and a skin biopsy sample was available for 10; all samples were positive for *R. sibirica mongolitimonae*. An acute-phase serum sample was also available for 13 patients; results of serologic testing were positive for only 2 (15%). A convalescent-phase serum sample was available from 5 patients; results were positive for 4 (80%). A skin biopsy sample was also positive for *R. sibirica mongolitimonae* by culture.

Statistical comparison of the 4 rickettsioses (Table) showed that a recent travel history was more common among patients with *R. africae* infection (p<0.001). *R. slovaca* infection was associated with absence of fever or rash (p<0.001 for each). Multiple eschars were associated with *R. africae* infection (p<0.001). An eschar on the neck was a characteristic of infection with *R. sibirica mongolitimonae* (p = 0.002); on the scalp, *R. slovaca* (p<0.001); on the trunk, *R. conorii* (p = 0.05); and on the lower limbs, *R. africae* (p<0.001). For patients with rope-like lymphangitis, the probability of *R. sibirica mongolitimonae* infection was 100% (p<0.001). Cervical lymphadenitis was associated with *R. slovaca* (p<0.001), inguinal lymphadenitis with *R. africae* (p<0.001), and axillary lymphadenitis with *R. sibirica mongolitimonae* infection (p = 0.01).

**Table:** Epidemiologic and clinical characteristics of the main spotted fever group rickettsioses identified at the Unité de Recherche sur les Maladies Infectieuses et Tropicales Émergentes, Marseilles, France, 2010–2014*

Characteristic	*Rickettsia africae*	*R. conorii*	*R. slovaca*	*R. sibirica mongolitimonae*
No. cases	36	21	14	20
Geographic location	Zimbabwe and South Africa	Algeria, France, Morocco, Portugal, South Africa	France	France, Spain
Median age ± SD (IQR), y	58 ± 12 (31–80)	53 ± 18 (10–80)	36 ± 23 (6–65)	43 ± 21 (2–70)
Female sex	14 (39)	7 (33)	9 (64)	8 (40)
Recent travel	36	17	0	1
Clinical signs				
Fever	35 (97)	21 (100)	5 (36)	20 (100)
Rash	24 (67)	20 (95)	3 (21)	19 (95)
Enlarged lymph nodes	15 (42)	3 (14)	14 (100)	12 (60)
Lymphadenopathy location				
Cervical	1 (3)	3 (14)	14 (100)	5 (25)
Inguinal	14 (39)	0	0	3 (15)
Axillary	0	0	0	4 (20)
Eschar	36 (100)	18 (86)	14 (100)	20 (100)
Multiple eschars	13 (36)	0	0	3 (15)
Eschar location				
Scalp	0	2 (10)	14 (100)	0
Lower limbs	32 (89)	3 (14)	0	7 (33)
Upper limbs	2 (6)	2 (10)	0	4 (20)
Trunk	2 (6)	5 (24)	0	3 (15)
Neck	0	0	0	4 (20)
Lymphangitis	0	0	0	7 (35)
Treatment (duration, d)				
Doxycycline	34 (1–20)	21 (7–21)	12 (1–7)	19 (7–14)
Amoxicillin	2 (7)	None	None	None
Pristinamycin	None	1 (7)	None	1 (7)
Azithromycin	None	None	2 (4)	None

*Values are no. (%) patients unless otherwise indicated. IQR, interquartile range.

## Conclusions  

*R. sibirica mongolitimonae* is considered a rare pathogen; only 30 cases of infection with this organism have been reported in Europe and Africa (Technical Appendix Table 2), of which 11 patients had lymphangitis, 27 inoculation eschars, and 18 a rash. In agreement with previous authors, we found that the most common signs of *R. sibirica mongolitimonae* infection were fever and rash. The addition of rope-like lymphangitis cases to those in the literature revealed that 17 (35%) of patients with *R. sibirica mongolitimonae* infection had this manifestation. Ramos et al. proposed that the term lymphangitis-associated rickettsiosis may be unwarranted for *R. sibirica mongolitimonae* infection because it is not found in all patients infected with this organism and because other rickettsioses produce lymphangitis (*14*). However, only *R. sibirica mongolitimonae* infection is associated with rope-like lymphangitis extending from the eschar to the draining lymph node; to our knowledge, only 1 case of mild, local, but not rope-like lymphangitis in a patient with *R. africae* infection has been described (*15*). In accordance with previous reports from France and Spain (*3*,*14*), we found that *R. sibirica mongolitimonae* infection was seasonal and that most cases occurred in the spring and summer.

Our strategy for diagnosing *Rickettsia* spp. infection on the basis of skin biopsy and cutaneous swab samples modified our knowledge of the epidemiology of SFG rickettsioses in France. We provide evidence that *R. sibirica mongolitimonae* infection is a frequent rickettsiosis, probably more frequent than *R. conorii* infection, which for decades has been considered the most common *Rickettsia* species in France.

Technical Appendix Epidemiologic, clinical, and microbiological characteristics associated Rickettsia sibirica mongolitimonae infection and reported cases, France, 2010­—2014.
