# Supplementary material for: Rickettsia sibirica mongolitimonae Infection, France, 2010–2014
Source: Emerg Infect Dis. 2016 May;22(5):880–2. doi: 10.3201/eid2205.141989 (PMC4861502; doi:10.3201/eid2205.141989)
Supplement: Technical Appendix — Epidemiologic, clinical, and microbiological characteristics associated Rickettsia sibirica mongolitimonae infection and reported cases, France, 2010­—2014. [file 14-1989-Techapp-s1.pdf]

Article DOI: http://dx.doi.org/10.3201/eid2205.141989

Rickettsia sibirica mongolitimonae Infection, France, 2010–2014

Technical Appendix

Technical Appendix Table 1. Epidemiologic, clinical, and microbiological characteristics associated with patients infected with *Rickettsia sibirica mongolitimonae*

Case	Sex	Age	Animal contact	Fever	Lymphadenopathy	Eschar	Laboratory data	Other	Serology (IgM/igG)		Treatment
									First serum	Convalescence	
1	M	50	Dogs	Yes (2 weeks)	No	Left Hand	ALT: 79 IU/L; AST: 70 IU/L; platelets: 59x 10^9/ L	Intensive care unit hospitalization	Negative	Positive (64/128)	Doxycycline (2 weeks)
2	M	36	Horses	Yes	Inguinal	Left foot	NP	Cough, rope-like lymphangitis	Negative	NR	Doxycycline (10 d)
3	M	30	No	Yes	No	Right leg	ALT: 61 IU/L; AST: 54 IU/L; platelets: 107x 10^9/ L	No	NR	NR	Doxycycline (1 week)
4	F	58	Dogs	Yes (10 d)	Cervical	Neck	ALT: 50 IU/L; AST: 47 IU/L; platelets: 137x 10^9/ L; Stp: 56 g/L	Confusion	Negative	NR	Doxycycline (1 week)
5	M	31	No	Yes	Axiliary and cervical	Back	NP	Rope like lymphangitis	NR	NR	Doxycycline (1 week)
6	M	17	Dogs	Yes	Cervical	Neck	NP	No	Negative	Positive (128/128)	Amox and then doxy
7	F	50	No	Yes	Inguinal	Thigh	NP	No	Negative	Negative	Doxycycline (1 week)
8 (1)	M	19	Dogs	Yes (4 d)	Cervical	Eyelid	NP	No	Negative	Positive (128/64)	Doxycycline (1 week)
9	F	29	No	Yes	Inguinal	Righ foot	NP	Rope like lymphangitis	NR	NR	Pristinamycine (7 d)
10	M	70	No	Yes	Axiliary	Left hypochondrium	NP	No	Negative	NR	Doxycycline (1 week)
11	M	67	Dogs	Yes (10 d)	Cervical	Neck	NP	No	Negative	NR	Doxycycline (10 d)
12	F	63	No	Yes (7 d)	Axiliary	Left Hand	NP	No	Negative	Positive (128/32)	Doxycycline (1 week)
13	F	40	No	Yes (15 d)	No	Right costal	ALT: 45 IU/L; AST: 49 IU/L; platelets: 97x 10^9/ L; Stp: 58 g/L	No	Positive (128/32)	NR	Doxycycline (2weeks)

Case	Sex	Age	Animal contact	Fever	Lymphadenopathy	Eschar	Laboratory data	Other	Serology (IgM/igG)		Treatment
									First serum	Convalescence	
14	F	76	No	Yes	No	Neck	NP	No	NR	NR	Doxycycline (1 week)
15	M	47	No	Yes (7 d)	Cervical	Left Hand	NP	Rope like lymphangitis	Negative	NR	Doxycycline (1 week)
16	M	57	No	Yes	No	3 eschars in the hand	NP	No	Negative	NR	Doxycycline
17 (2)	F	67	No	Yes	No	Left leg	NP	Rope like lymphangitis	Positive (64/64)	NR	Doxycycline (1 week)
18	M	28	No	Yes	No	Chest	ALT: 55 IU/L; AST: 48 IU/L; platelets: $115x\ 10^9/\ L$	Rope like lymphangitis	NR	NR	Doxycycline (1 week)
19	M	2	No	Yes	No	Ear	NP	No	NR	NR	Doxycycline (1 week)
20	F	10	No	Yes	Popliteal	Left leg	NP	Rope like lymphangitis	NR	NR	Doxycycline (1 week)

*NR, not received; NP, not provided; ALT, alanine aminotransferase; AST, aspartate aminotransferase; Stp, serum total protein.

Technical Appendix Table 2. Previous reported cases of *Rickettsia sibirica mongolitimonae**

Year- Reference	Number of cases	Country	Rope-like lymphangitis (No. cases, %)	Ecshar (No. cases, %)	Rash (No. cases, %)	Other
1996 - (*1*)	1	France	No	Yes (1, 100%)	Yes (1, 100%)	
2000 - (*2*)	1	France	Yes (1, 100%)	Yes (1, 100%)	No	
2004 - (*3*)	1	South Africa	Yes (1, 100%)	Yes (1, 100%)	No	
2005 - (*4*)	7	France	Yes (3, 43%)	Yes (6, 86%)	Yes (6, 86%)	
2005 - (*5*)	1	Greece	No	Yes (1, 100%)	Yes (1, 100%)	
2006 - (*6*)	1	Portugal	No	Yes (1, 100%)	Yes (1, 100%)	
2008 - (*7*)	1	Spain	No	Yes (1, 100%)	No	
2008 - (*8*)	1	Portugal	Yes (1, 100%)	Yes (1, 100%)	Yes (1, 100%)	
2008 - (*9*)	1	France	Yes (1, 100%)	Yes (1, 100%)	Yes (1, 100%)	Retinal vasculitis
2010 - (*10*)	1	Egypt	No	Yes (1, 100%)	No	
2011 - (*11*,*12*)	2	Spain	No	Yes (2, 100%)	Yes (1, 100%)	
2012 - (*13*)	2	Algeria	NP	NP	NP	
2012 - (*11*)	1	Spain	No	Yes (1, 100%)	Yes (1, 100%)	septic shock
2013 - (*14*)	6	Spain	Yes (3, 50%)	Yes (6, 100%)	Yes (4, 67%)	
2013 - (*15*)	2	France	Yes (1, 50%)	Yes (2, 100%)	Yes (1, 50%)	
2014 - (*16*)	1	France	No	Yes (1, 100%)	No	
Total	30		11 (37%)	27 (90%)	18 (60%)	

References

1. Raoult D, Brouqui P, Roux V. A new spotted-fever-group rickettsiosis. Lancet. 1996;348:412. PubMed http://dx.doi.org/10.1016/S0140-6736(05)65037-4

2. Fournier PE, Tissot-Dupont H, Gallais H, Raoult D. *Rickettsia mongolotimonae*: a rare pathogen in France. Emerg Infect Dis. 2000;6:290–2. PubMed http://dx.doi.org/10.3201/eid0603.000309

3. Pretorius AM, Birtles RJ. *Rickettsia mongolotimonae* infection in South Africa. Emerg Infect Dis. 2004;10:125–6. PubMed http://dx.doi.org/10.3201/eid1001.020662

4. Fournier PE, Gouriet F, Brouqui P, Lucht F, Raoult D. Lymphangitis-associated rickettsiosis, a new rickettsiosis caused by *Rickettsia sibirica mongolotimonae*: seven new cases and review of the literature. Clin Infect Dis. 2005;40:1435–44. PubMed http://dx.doi.org/10.1086/429625

5. Psaroulaki A, Germanakis A, Gikas A, Scoulica E, Tselentis Y. Simultaneous detection of "*Rickettsia mongolotimonae*" in a patient and in a tick in Greece. J Clin Microbiol. 2005;43:3558–9. PubMed http://dx.doi.org/10.1128/JCM.43.7.3558-3559.2005

6. de Sousa R, Barata C, Vitorino L, Santos-Silva M, Carrapato C, Torgal J, et al. *Rickettsia sibirica* isolation from a patient and detection in ticks, Portugal. Emerg Infect Dis. 2006;12:1103–8. PubMed http://dx.doi.org/10.3201/eid1207.051494

7. Aguirrebengoa K, Portillo A, Santibanez S, Marin JJ, Montejo M, Oteo JA. Human *Rickettsia sibirica mongolitimonae* infection, Spain. Emerg Infect Dis. 2008;14:528–9. PubMed http://dx.doi.org/10.3201/eid1403.070987

8. de Sousa R, Duque L, Anes M, Pocas J, Torgal J, Bacellar F, et al. Lymphangitis in a Portuguese patient infected with *Rickettsia sibirica*. Emerg Infect Dis. 2008;14:529–30. PubMed http://dx.doi.org/10.3201/eid1403.070680

9. Caron J, Rolain JM, Mura F, Guillot B, Raoult D, Bessis D. *Rickettsia sibirica* subsp. *mongolitimonae* infection and retinal vasculitis. Emerg Infect Dis. 2008;14:683–4. PubMed http://dx.doi.org/10.3201/eid1404.070859

10. Socolovschi C, Barbarot S, Lefebvre M, Parola P, Raoult D. *Rickettsia sibirica mongolitimonae* in traveler from Egypt. Emerg Infect Dis. 2010;16:1495–6. PubMed http://dx.doi.org/10.3201/eid1609.100258

11. Ibarra V, Portillo A, Palomar AM, Sanz MM, Metola L, Blanco JR, et al. Septic shock in a patient infected with *Rickettsia sibirica mongolitimonae*, Spain. Clin Microbiol Infect. 2012;18:E283–5. PubMed http://dx.doi.org/10.1111/j.1469-0691.2012.03887.x

12. Fleta-Asín B, Alonso-Castro L, Jado-Garcia I, Anda-Fernandez P. Detection by polymerase chain reaction of *Rickettsia sibirica mongolotimonae* in the skin biopsy of a rash: a case report. Enferm Infecc Microbiol Clin. 2011;29:778–9. PubMed

13. Mokrani K, Tebbal S, Raoult D, Fournier PE. Human rickettsioses in the Batna area, eastern Algeria. Ticks Tick Borne Dis. 2012;3:364–6. PubMed http://dx.doi.org/10.1016/j.ttbdis.2012.10.017

14. Ramos JM, Jado I, Padilla S, Masia M, Anda P, Gutierrez F. Human infection with *Rickettsia sibirica mongolitimonae*, Spain, 2007–2011. Emerg Infect Dis. 2013;19:267–9. PubMed http://dx.doi.org/10.3201/eid1902.111706

15. Edouard S, Parola P, Socolovschi C, Davoust B, La Scola B, Raoult D. Clustered cases of *Rickettsia sibirica mongolitimonae* infection, France. Emerg Infect Dis. 2013;19:337–8. PubMed http://dx.doi.org/10.3201/eid1902.120863

16. Solary J, Socolovschi C, Aubry C, Brouqui P, Raoult D, Parola P. Detection of *Rickettsia sibirica mongolitimonae* by using cutaneous swab samples and quantitative PCR. Emerg Infect Dis. 2014;20:716–8. PubMed http://dx.doi.org/10.3201/eid2004.130575
